# From skin lesions to multi-organ involvement: Organ tropism and pathogenesis of mpox virus

**DOI:** 10.1016/j.isci.2025.114209

**Published:** 2025-11-25

**Authors:** Anna-Lena Rupp, Jo Paul Meister, Julian Schulze Zur Wiesch, Toni Luise Meister

**Affiliations:** 1 Institute for Infection Research and Vaccine Development (IIRVD), University Medical Centre Hamburg-Eppendorf, 20246 Hamburg, Germany; 2 Department for Clinical Immunology of Infectious Diseases, Bernhard Nocht Institute for Tropical Medicine, 20359 Hamburg, Germany; 3 German Centre for Infection Research (DZIF), Partner Site Hamburg-Lübeck-Borstel-Riems, 20246 Hamburg, Germany; 4 Department of Thoracic Surgery, Thoraxklinik, University of Heidelberg, 69126 Heidelberg, Germany; 5 Centre of Internal Medicine, University Medical Centre Hamburg-Eppendorf, 20246 Hamburg, Germany

**Keywords:** Virology, Pathology, Pathogenic organism

## Abstract

Mpox virus (MPXV), the causative agent of mpox, is a re-emerging zoonotic orthopoxvirus. Until 2022, infections were largely confined to endemic regions in Central and West Africa. However, following phylogenetic divergence and a subsequent global outbreak in 2022 caused by clade IIb, distinct from the clade I-driven resurgence in the Democratic Republic of the Congo in 2024, MPXV has emerged as a significant global health concern. While mpox is primarily characterized by its distinctive skin lesions, growing clinical and experimental evidence suggests that MPXV can replicate and disseminate to further organ systems, potentially contributing to disease severity. Despite this growing knowledge, the mechanisms underlying MPXV organ tropism and pathogenesis remain poorly understood, posing ongoing challenges for research and clinical management. This review summarizes the latest clinical, pathological, and experimental findings to provide an updated understanding of MPXV tropism and pathogenesis.

## Graphical abstract

**Figure:**
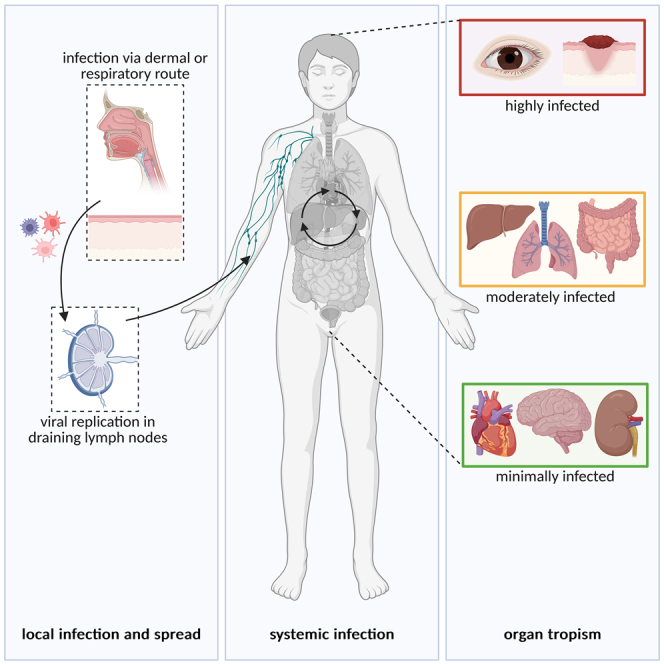

## Introduction

Mpox is a re-emerging zoonotic disease caused by the mpox virus (MPXV). MPXV is a DNA virus of the *Orthopoxvirus* genus, closely related to variola virus, the causative agent of smallpox.^1^ MPXV was first identified in monkeys in 1958, with the first human case reported in the Democratic Republic of the Congo (DRC) in 1970.^2^^,^^3^ Human infections result from zoonotic spillover or direct human-to-human transmission via close contact by skin lesions, respiratory secretions, contaminated materials, or, more recently, sexual contact.^4^ Before 2022, most cases were confined to endemic regions of Central and West Africa, with sporadic international cases due to travel or importation of infected animals.^5^^,^^6^^,^^7^^,^^8^^,^^9^ This changed with the global outbreak in 2022, which spread across over 100 countries and prompted the World Health Organization (WHO) to declare a Public Health Emergency of International Concern (PHEIC). Although the PHEIC was lifted in 2023, it was reinstated in 2024 following a resurgence in the DRC.^10^ Between January 2022 and March 2025, these outbreaks caused nearly 140,000 laboratory-confirmed cases and over 300 deaths worldwide, highlighting the growing public health impact of MPXV.^11^ These outbreaks were driven by distinct viral lineages. MPXV is genetically divided into two major clades, known as clade I and clade II, with clade I being associated with more severe disease, higher transmissibility, and mortality.^12^^,^^13^^,^^14^ Further phylogenetic divergence within each clade has led to the identification of subclades, clade Ia and IIa, associated with sporadic outbreaks in endemic regions, and Ib and IIb, which are responsible for the 2024 and 2022 outbreaks, respectively.^15^^,^^16^ Infections with the initial strains of MPXV primarily resulted from zoonotic spillovers with limited secondary transmission. In contrast, both the 2022 clade IIb outbreak and the 2024 clade Ib outbreak in the DRC were characterized by sustained human-to-human transmission through sexual and non-sexual contact.^16^^,^^17^^,^^18^ These widespread outbreaks highlight the urgent need for effective preventive and therapeutic measures. Currently, the treatment of mpox primarily consists of supportive care and pain management. In severe or high-risk cases, antivirals approved for the treatment of smallpox have been applied, especially tecovirimat, and also brincidofovir or cidofovir in some cases.^19^^,^^20^^,^^21^^,^^22^ Three vaccines originally designed for smallpox are currently licensed to prevent MPXV infection: ACAM2000, a replication-competent live vaccine; JYNNEOS (also known as Imvamune or MVA-BN), a third-generation, non-replicating modified vaccinia Ankara vaccine^23^; and LC16m8, a live-attenuated vaccinia virus strain.^24^

## MPXV virus-host interactions

MPXV is a member of the *Orthopoxvirus* genus, which comprises large enveloped viruses. Mature MPXV virions are ovoid or brick-shaped and measure 200–250 nm in diameter.^25^ They contain a double-stranded DNA genome of ∼197 kb that encodes around 190 proteins.^26^^,^^27^ Like all orthopoxviruses, MPXV replicates entirely within the host cell cytoplasm.^28^ The viral life cycle begins with attachment to host cells. To date, no specific cellular receptor that mediates orthopoxvirus entry has been identified. Instead, viral entry is proposed to occur via widely expressed and highly conserved cell surface molecules, such as glycosaminoglycans or other extracellular matrix components.^29^^,^^30^^,^^31^^,^^32^ A viral protein proposed to be involved in the entry process is MPXV A29, which was demonstrated to bind GAGs, including heparin and chondroitin sulfate/dermatan sulfate.^33^ Furthermore, a ganglioside-binding motif has been identified in the MPXV surface-binding protein E8L.^34^^,^^35^ Following attachment and entry, the viral core is delivered into the cytoplasm, where the genome and viral proteins are released upon uncoating. These factors facilitate viral DNA replication and transcription, followed by the translation of early, intermediate, and late viral proteins.^36^^,^^37^ Subsequent assembly of new virions begins with the formation of nucleocapsids and the incorporation of viral glycoproteins to generate intracellular mature virus (IMV) with a single lipid membrane.^38^^,^^39^ IMVs can be released through cell lysis, or they can be further enveloped by membranes derived from the *trans*-Golgi network or endosomes.^40^^,^^41^ The virions are then transported to the cell surface and released as extracellular enveloped virus (EEV), a form particularly important for viral dissemination.^42^^,^^43^

These molecular features underlie a broad cell and tissue tropism of MPXV, both *in vitro* and *in vivo*. This is potentially facilitated by its ability to bind to ubiquitous attachment factors, enabling the virus to enter many cell types. However, successful viral replication depends on intracellular events that occur after binding and entry of the virus.^44^^,^^45^^,^^46^ In addition to its wide cellular tropism, MPXV exhibits a broad host tropism, infecting various mammalian species. These include rodents, such as mice, rabbits, squirrels, and prairie dogs, as well as non-human primates (NHPs).^2^^,^^47^^,^^48^^,^^49^^,^^50^ Despite being named MPXV, NHPs, as well as humans, are considered incidental hosts.^51^ The definitive natural reservoir remains to be identified, although rodents are considered the most likely candidates.^9^ This wide host range distinguishes MPXV from other orthopoxviruses, such as variola virus, which only infects humans.^52^

In summary, the determinants of the broad tissue and cell tropism of MPXV remain incompletely understood. Current insights largely come from animal models and clinical studies, which will be reviewed in the following sections.

## Pathogenesis, clinical presentation, and experimental observation

**Figure 1:**
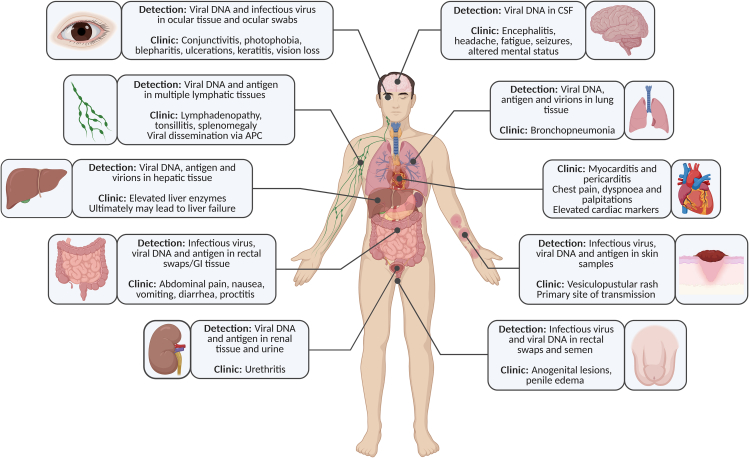
Summary of the tissue tropism, pathophysiological changes, and clinical manifestations associated with MPXV infection. The figure highlights organ systems and anatomical sites where MPXV has been detected, indicating viral replication or immune response involvement. Clinical signs and symptoms are linked to affected tissues where possible. This overview is based on current histopathological, molecular, and clinical findings related to human MPXV infections.

MPXV enters the human body via the respiratory epithelium, through inhalation or through direct contact with infectious material via the dermal route. From the initial site of infection, the virus is thought to spread to draining lymph nodes via infected immune cells, where it undergoes replication, resulting in a low-grade primary viremia. This facilitates subsequent dissemination to larger organs, where further viral amplification occurs, leading to a secondary viremia that enables systemic spread to distant organs.^19^^,^^53^ The incubation period between infection and symptom onset ranges from 5-21 days.^54^ This is followed by a prodromal phase of 2–4 days, characterized by systemic symptoms including fever, myalgia, headache, and notably, lymphadenopathy.^55^^,^^56^^,^^57^^,^^58^^,^^59^^,^^60^^,^^61^ Subsequently, patients develop a characteristic vesiculopustular rash that persists for 2–4 weeks.^61^ While mpox is usually self-limiting, immunocompromised patients, children, and pregnant individuals are at a higher risk of complications and severe outcomes.^62^ Although cutaneous lesions remain the hallmark of MPXV infection, emerging evidence suggests that the virus can disseminate beyond the skin to multiple organ systems, potentially contributing to disease severity and the development of complications (Figure 1).

### Skin

Mpox is characterized by skin lesions that progress through distinct stages. Starting as maculopapular lesions, they evolve into vesicles, pustules, and crusts, which fall off once new skin has formed. The lesions remain infectious throughout this process until the skin has healed.^19^^,^^63^ Skin lesions occur in almost all documented mpox cases and can spread widely across the body.^16^^,^^64^

Clinical presentation, however, varies depending on the viral clade. Infections with clades I and IIa are more commonly associated with a pronounced prodromal phase, marked by symptoms such as fever, headache, and lymphadenopathy prior to rash onset.^65^^,^^66^ Outbreaks in endemic regions, typically linked to these clades, also feature widespread, synchronous lesion development, often affecting the face and extremities.^55^^,^^56^ In contrast, the 2022 global outbreak caused by clade IIb displayed notable differences. Many patients presented directly with skin or mucosal lesions, probably due to the mode of acquisition, and prodromal symptoms were absent or mild in a substantial proportion of cases.^65^^,^^66^ Lesions were usually fewer, developed asynchronously, and predominantly appeared in the anogenital region.^57^^,^^65^^,^^66^^,^^67^^,^^68^^,^^69^^,^^70^ Similarly, infections with clade Ib during the 2024 outbreak frequently involved genital lesions, reinforcing the possibility that sexual transmission may influence lesion distribution and disease presentation.^71^

These patterns of variation are not limited to unvaccinated individuals. Breakthrough infections following vaccination, or vaccination used as post-exposure prophylaxis, were associated with milder disease, with affected patients exhibiting less-pronounced prodromal symptoms and developing fewer skin lesions compared with unvaccinated individuals. However, differences in clinical presentation between clades during natural infection were similarly observed among breakthrough cases,^72^ indicating that viral genetic background continues to influence disease characteristics regardless of vaccination status.

Histological analysis of skin biopsies from immunocompromised patients revealed extensive lesions affecting both the epidermis and dermis. Viral antigen and DNA have been detected in epithelial cells at sites of vesicle formation and along ulcer margins.^73^^,^^74^ Furthermore, MPXV virions have been visualized by transmission electron microscopy (TEM) in skin tissue samples from MPXV-infected patients.^61^^,^^73^^,^^75^^,^^76^^,^^77^ The cellular targets of MPXV within the skin are proposed to include keratinocytes and fibroblasts. This is supported by experimental *in vitro* infections of both immortalized and primary human keratinocytes,^78^^,^^79^ as well as immortalized fibroblasts.^75^ These findings are further corroborated by *in vivo* data from experimentally infected cynomolgus monkeys, which demonstrated detection of MPXV in keratinocytes, ranging from the basal to granular layers of the epidermis, as well as in dermal fibroblasts.^53^ Consistently, *in vivo* imaging in BALB/c mice revealed extensive viral replication in the skin of the feet and tails, while PCR confirmed viral DNA in skin samples from C57BL/6 mice.^80^^,^^81^

Consistent with the significant cutaneous involvement, skin samples have been shown to harbor the highest viral loads, highlighting the skin as a critical site for viral replication and transmission.^82^^,^^83^

### Lymphatic system

A distinguishing symptom of mpox, in contrast to other orthopoxviruses such as variola virus, is lymphadenopathy.^84^ It typically affects the maxillary, supraclavicular, axillary, cervical, or inguinal lymph nodes and has been reported in up to almost every case in some outbreaks, although prevalence varies considerably across studies.^16^^,^^55^^,^^68^^,^^70^^,^^85^^,^^86^^,^^87^ Although less frequently reported, other lymphatic manifestations include splenomegaly and tonsillitis.^68^^,^^87^^,^^88^^,^^89^ Further insight into lymphatic involvement comes from autopsy findings in severe cases among immunocompromised individuals. These revealed necrotizing lesions in lymph nodes, spleen, and bone marrow, marked by the detection of viral antigen and DNA.^73^ These clinical findings are corroborated by experimental models. In MPXV-infected CAST/EiJ mice, viral DNA and infectious virus were detected in inguinal lymph nodes and spleen.^90^^,^^91^^,^^92^^,^^93^^,^^94^ Marmosets infected intravenously with MPXV developed pronounced lymphadenopathy in axillary, inguinal, or mandibular lymph nodes, along with histopathological lesions in the lymph nodes, spleen, and bone marrow.^50^ Similarly, cynomolgus macaques exposed to aerosolized MPXV exhibited generalized lymphadenopathy and necropsy studies showed widespread lesions in lymph nodes, tonsils, spleen, bone marrow, and thymus.^14^^,^^53^^,^^95^^,^^96^ Immunohistochemistry and viral isolation confirmed the presence of viral DNA and replication-competent virus in lymphoid tissues, including lymph nodes, spleen, and tonsils.^53^^,^^96^ TEM and antigen detection identified macrophages and dendritic cells as common viral targets within different lymphoid tissues.^53^ These findings, alongside the observation that Kupffer cells were preferentially infected in the liver, support the notion that MPXV targets the mononuclear phagocyte system.^53^ These findings align with the hypothesis that antigen-presenting cells may transport the virus to draining lymph nodes, facilitating systemic dissemination via the lymphatic system.^1^^,^^19^ Importantly, structural damage to lymphoid organs could impair the host’s immune response, increasing susceptibility to secondary infections. This is supported by an observed reduction in the number of various immune cells and lymphoid depletion in multiple lymphatic tissues including lymph nodes, spleen, and tonsils in cynomolgus macaques following MPXV infection.^95^^,^^96^

Taken together, the clinical and experimental evidence underscores the central role of the lymphatic system in MPXV pathogenesis concerning viral replication, distribution, and immune response.

### Bloodstream

MPXV pathogenesis involves a primary viremia following replication in lymphatic tissue, followed by a secondary viremia driven by viral replication in larger organs.^19^ Patients in whom viremia was detected at diagnosis showed a higher frequency of systemic symptoms and more disseminated lesions than non-viremic patients, suggesting that bloodstream dissemination contributes to mpox pathogenesis.^97^

Despite these viremic phases, MPXV DNA levels in blood are generally low and are detected less frequently than in other clinical specimens, such as skin lesions or oropharyngeal swabs.^82^^,^^83^^,^^98^^,^^99^ When comparing MPXV detection across blood fractions, viral DNA was more frequently detected in the plasma and at higher copy numbers than in serum or whole blood.^99^ Importantly, no infectious virus has been isolated from blood and viral clearance occurs more rapidly in blood than in other sample types.^83^^,^^99^ Consistent with these findings, no cases of MPXV transmission via blood transfusion have been reported to date, although data on the risk of blood-borne transmission remain limited.^100^^,^^101^

These clinical observations are supported by experimental data from NHPs, where viral DNA was detectable in blood as early as day 2 post-inoculation, with peak levels typically occurring between days 7 and 10 depending on the infection route, dose, and viral strain.^96^^,^^102^^,^^103^^,^^104^ However, infectious virus could not be recovered from whole blood at any time point,^96^^,^^103^ a finding that contradicts results obtained in mouse models. In fact, viral DNA and infectious virus were detected in the blood of MPXV-infected C57BL/6, BALB/c, and CAST/EiJ mice.^80^^,^^90^ Notably, clade-specific differences in viral loads have been reported in NHPs, with approximately 10-fold higher viral genome numbers in whole blood of animals infected with clade Ia compared to clade IIa.^14^

With regard to infection of cellular blood components, MPXV DNA has not been detected in erythrocytes from a blood donation of an mpox patient.^100^ In contrast, a recent preprint demonstrated that peripheral blood mononuclear cells (PBMCs) are susceptible to MPXV infection. Monocytes, cycling natural killer (NK) cells, and regulatory CD4^+^ T cells were shown to be infected, with monocytes displaying viral RNA at earlier time points.^105^ Notably, infectious virus could be recovered from PBMCs and buffy coats of NHPs infected via the intranasal or subcutaneous routes, confirming that blood cells support MPXV infection *in vivo.*^106^ Consistent with this observation, studies in intravenously infected NHPs confirmed granulocytes and monocytes as the predominant target cell types, while leukocytes, including CD20^+^ B cells, NK cells, CD4^+^ T cells, and CD8^+^ T cells, also stained positive for pox antigen. Importantly, the frequency and timing of pox-antigen-positive immune cells correlated with disease progression, as animals with earlier detection and higher frequencies tended to develop more severe disease and reach a moribund endpoint sooner.^107^

Together, these findings highlight the potential involvement of the bloodstream and its circulating immune cell populations in viral dissemination, underscoring the need for further research on the role of hematogenous spread in MPXV pathogenesis.

### Central nervous system

The broad clinical spectrum of MPXV infection also includes neurological complications, which range from mild to life-threatening. Common symptoms such as headache, myalgia, and fatigue are frequently reported, while more severe neurological manifestations, such as encephalitis, seizures, and altered mental status, occur in a smaller subset of cases.^108^^,^^109^ These serious complications are estimated to affect approximately 3% of infected individuals and can be fatal.^55^^,^^108^^,^^110^^,^^111^ The potential for direct central nervous system (CNS) involvement is supported by both human and animal studies. In humans, MPXV DNA has been detected in cerebrospinal fluid using PCR, providing direct evidence of CNS infection.^112^ Moreover, multiple animal studies have confirmed the neuroinvasive potential of MPXV, as viral DNA and infectious particles have been found in the brain tissue of various rodent species and rhesus macaques and their fetuses.^48^^,^^90^^,^^91^^,^^113^^,^^114^^,^^115^^,^^116^^,^^117^^,^^118^^,^^119^ These findings indicate that MPXV may be capable of crossing the blood-brain barrier (BBB). The exact mechanism by which MPXV enters the CNS remains unclear; however, two primary routes have been proposed based on animal models. One is the olfactory route, in which the virus infects the olfactory epithelium and travels to the brain via the olfactory nerve. The second proposed route is hematogenous spread, where infected leukocytes cross the BBB, allowing the virus to access neural tissue.^109^

Emerging evidence highlights the susceptibility of human neural cells to MPXV infection. *In vitro* studies have shown that human induced pluripotent stem cell (iPSC)-derived neural progenitor cells and astrocytes support productive viral replication.^120^ This has been further corroborated by findings from *ex vivo* human brain tissue and neural organoids, where MPXV infection has also been demonstrated.^121^^,^^122^ These models revealed that astrocytes and microglia support more efficient MPXV replication compared to neurons, although all three cell types are permissive to infection. Within human neural tissue, MPXV appears to spread primarily via direct cell-to-cell transmission, including along axons and synaptic connections.^122^ Recent studies have also shed light on MPXV pathogenesis in the CNS, showing that the virus triggers inflammation-induced cell death of astrocytes via gasdermin B-mediated pyroptosis. Given the central role of astrocytes in maintaining CNS homeostasis, their targeted destruction may substantially contribute to MPXV-associated neuropathology.^123^ In addition, infected neurons have been observed to form neuritic beads, a hallmark of cellular injury seen in conditions such as ischemia,^124^ Alzheimer disease,^125^^,^^126^ and epilepsy.^127^ This structural degeneration is followed by neuronal death, which may underlie some of the neurological symptoms observed in MPXV-infected individuals.^122^

Taken together, these findings suggest that MPXV is not only capable of infecting a wide range of human neural cell types but also induces direct and potentially lasting damage to the CNS, underscoring its neurovirulent potential.

### Eyes

Ocular manifestations associated with MPXV infection range from mild to sight-threatening conditions. The most common presentation is conjunctivitis, reported with a pooled prevalence of approximately 12%. At the same time, other symptoms such as photophobia, blepharitis, and focal lesions of the periocular skin, conjunctiva, and cornea appear less frequently.^128^ Severe complications, including corneal ulcerations, keratitis, and eventual vision loss, have been documented in 3%–4% of individuals infected with MPXV.^55^^,^^56^^,^^58^^,^^68^^,^^88^^,^^128^ However, the prevalence and severity of ocular involvement have varied significantly between outbreaks, with higher rates generally reported before 2022, suggesting ocular symptoms to be more common in patients infected through zoonotic transmission of animal-derived MPXV.^88^^,^^128^ It is thought that these symptoms primarily result from autoinoculation rather than from dissemination through systemic viremia.^128^^,^^130^

These clinical observations are supported by the detection of viral antigen and DNA in the tissues of ocular lesion sites in immunocompromised patients.^73^ MPXV DNA has also been detected in tear fluid, eyelid swabs, and conjunctival swabs from patients with ocular involvement.^129^^,^^130^^,^^131^^,^^132^^,^^133^^,^^134^^,^^135^ Notably, replication competent virus has been isolated from conjunctival swabs, underscoring the potential risk of transmission via ocular secretions.^130^

Similar findings have been reported in animal studies. Viral DNA and shedding has been detected in ocular swabs from infected prairie dogs.^136^ Moreover, MPXV DNA was identified in the eye tissue of fetal demise cases of rhesus macaques.^119^ Additionally, cynomolgus monkeys infected with aerosolized MPXV developed necrotizing conjunctivitis, with detection of viral antigen within conjunctival epithelial cells and fibroblasts.^53^

These findings collectively highlight the eye as a relevant site of MPXV replication. The detection of viral DNA, antigen, and even replication-competent virus in ocular tissues and secretions, across both human and animal studies, underscores the clinical and epidemiological significance of ocular involvement in mpox. While animal models have provided valuable insights into ocular pathology, the current lack of *in vitro* studies limits our understanding of the underlying cellular mechanisms. Future research using human ocular cell models is essential to better elucidate MPXV tropism, pathogenesis, and transmission risk in the ocular compartment.

### Cardiovascular system

One rare but significant complication of MPXV infection is cardiovascular involvement, which has only been reported in around 15 cases to date.^137^^,^^138^ Cardiovascular manifestations associated with MPXV infection vary in type and severity, with chest pain reported as the most common symptom. Other symptoms include chest tightness, dyspnea, palpitations, and reduced exercise capacity. Based on elevated cardiac markers such as troponin, creatine kinase, and C-reactive protein, as well as observations from electrocardiography and cardiac magnetic resonance imaging, the most prevalent complications were acute myocarditis and pericarditis.^137^^,^^138^^,^^139^ These conditions can result in pericardial effusion, arrhythmia, and heart failure. In some cases, this requires intensive care treatment and mechanical cardiovascular support.^140^

Cardiac involvement, particularly viral myocarditis, is a well-documented complication of several viral infections. The most common form of viral myocarditis is lymphocytic myocarditis, which is characterized by inflammatory infiltrates and myocyte necrosis.^141^ Cardiac complications associated with orthopoxviruses gained broad attention during the smallpox vaccination campaigns, when cases of post-vaccinal myocarditis and pericarditis were reported.^142^ The pathophysiology behind cardiac involvement following smallpox vaccination remains unclear. The absence of detectable virus in myocardial tissue in patients with post-vaccination myocarditis suggests an immune-mediated pathogenesis.^142^^,^^143^ However, some hemorrhagic smallpox patients demonstrated myocardial and endocardial bleeding, consistent with observations from a macaque model of hemorrhagic smallpox, where late-onset myocarditis was associated with lymphohistiocytic infiltrates containing virus-positive cells.^144^ These findings suggest that orthopoxviruses may have a broader capacity to affect cardiac tissue. Supporting this, MPXV DNA has been detected in the heart tissue of cynomolgus macaques following infection with aerosolized MPXV.^96^ Furthermore, the presence of MPXV antigen, viral DNA, and replication-competent virus was confirmed in heart tissue samples taken from MPXV-infected BALB/c and CAST/EiJ mice.^81^^,^^90^

Together, these findings raise the possibility that MPXV-associated myocarditis and pericarditis may result from direct viral invasion, an immune-mediated response, or a combination of both.

### Respiratory system

Respiratory involvement is a common aspect of MPXV infection, impacting both the upper and lower respiratory tracts. Among the most frequently reported symptoms are sore throat, with a pooled prevalence of 22%, and cough, occurring in approximately 16% of cases.^145^ Other symptoms include a runny nose, dyspnea, and wheezing. In more severe cases, respiratory distress or bronchopneumonia may develop.^55^^,^^70^^,^^85^^,^^146^^,^^147^^,^^148^^,^^149^ Lesions on the mucous membranes of the oral cavity are also commonly observed and can cause significant discomfort.^57^^,^^65^^,^^70^^,^^85^^,^^149^ Severe or fatal cases, particularly in immunocompromised patients, often exhibit substantial pulmonary involvement, such as bronchopneumonia. Pneumonia can be accompanied by pleural effusion and severe respiratory failure requiring mechanical ventilation subsequently increasing the risk of secondary nosocomial infections.^150^^,^^151^ Histological analyses of these fatal cases have identified MPXV antigen and DNA within lung tissues, with virions specifically localized in respiratory epithelial cells.^73^^,^^151^ Additionally, the presence of MPXV in the respiratory tract has been confirmed through the detection of viral DNA in saliva and throat swabs.^70^^,^^83^^,^^98^^,^^99^^,^^152^^,^^153^^,^^154^^,^^155^ In some cases, infectious virus has been successfully isolated from these specimens, indicating the potential for viral shedding via the upper respiratory tract.^154^

Experimental data from animal models further support the role of the respiratory tract in both MPXV pathogenesis and transmission. In several mouse models, viral antigen, MPXV DNA, and infectious virus have been detected in the lungs following experimental infection.^81^^,^^90^^,^^94^^,^^156^^,^^157^ Similarly, cynomolgus macaques infected with aerosolized MPXV demonstrated viral antigen, detectable viral DNA, and infectious virus in lung tissue, often accompanied by bronchopneumonia.^53^^,^^95^^,^^96^^,^^158^^,^^159^ Further respiratory lesions observed in this model include tracheitis, laryngitis, and mediastinitis.^53^ Moreover, MPXV shedding in nasal and throat swabs has been detected in infected NHPs.^103^ Together with evidence of airborne transmission under experimental conditions, these findings suggest that human-to-human transmission through respiratory droplets or aerosols is possible. However, it is likely less efficient than transmission via direct skin contact.^53^^,^^158^^,^^160^ Furthermore, the route of infection has been shown to influence both respiratory involvement and disease progression.^159^ Cynomolgus macaques infected via the intrabronchial route developed more severe pneumonia than those infected intravenously. However, key disease events, such as the onset of fever, lesion development, peak viremia, viral shedding, and meeting endpoint criteria occurred later in the course of disease.^159^

In addition to clinical and *in vivo* data, *in vitro* models offer valuable insight into the susceptibility of respiratory tissues to MPXV infection and provide a platform for future research. Human airway epithelial cell lines Calu-3 and A549, as well as primary human alveolar epithelial cells (hAECs) cultured at the air-liquid interface have been shown to be susceptible to MPXV infection.^161^^,^^162^^,^^163^ Infection of hAECs has been observed to reduce transepithelial electrical resistance and disrupt tight junction proteins, thereby compromising the integrity of the alveolar epithelial barrier.^163^ This disruption likely contributes to lung pathology during MPXV infection. Understanding these mechanisms is essential for developing targeted interventions to mitigate respiratory involvement and reduce the risk of airborne spread in humans.

### Gastrointestinal tract

The gastrointestinal (GI) tract represents another organ system that is commonly affected during MPXV infection. Frequently reported GI manifestations include abdominal pain (9%), nausea (10%), vomiting (12%), diarrhea (5%), rectal pain (11%), proctitis (11%), and rectal bleeding (12%).^71^^,^^164^^,^^165^ However, the reported prevalence varies considerably between studies, likely reflecting differences in patient populations and study designs. Notably, the incidence of proctitis was higher among men who engage in anal-receptive sex, indicating that direct inoculation of the rectal mucosa may contribute to pathogenesis.^66^^,^^166^ Less common manifestations include proctalgia and rare cases of rectal perforation.^65^^,^^164^^,^^167^ Additionally, in fatal mpox cases among AIDS patients, nodular ulcerative colitis has been documented.^151^

In general, GI involvement has been observed across multiple mpox outbreaks, regardless of the route of transmission. During the 2003 US outbreak, where infections were primarily acquired via animal bites or scratches, GI symptoms were commonly reported.^146^ Similar findings have emerged in recent outbreaks driven by close physical and sexual contact.^60^^,^^65^ These observations suggest that GI manifestations are a consistent feature of MPXV infection, independent of the route of transmission.

MPXV infection of the GI tract is further supported by detection of viral DNA in rectal swabs of patients both with and without proctitis.^83^^,^^98^^,^^154^^,^^155^^,^^166^^,^^168^^,^^169^^,^^170^^,^^171^^,^^172^ Moreover, replication-competent MPXV could be isolated from these samples.^171^^,^^172^ Autopsy tissue from deceased immunocompromised patients revealed mucocutaneous lesions in the GI tract, with viral antigen and DNA localized in epithelial cells at sites of vesicle formation and the acanthotic margins of ulcers.^73^

GI involvement is further corroborated by experimental studies in animal models. Infected BALB/c and CAST/EiJ mice showed viral antigen, DNA, and replication-competent virus in the stomach and intestines, with particularly high viral loads in the intestines compared to other organs.^81^^,^^90^ Furthermore, MPXV infection of cynomolgus monkeys revealed a high frequency of digestive tract lesions.^53^ Cynomolgus monkeys infected with aerosolized MPXV developed colitis and gastritis, with lesions predominantly affecting the distal colon and rectum. Immunohistochemical analysis confirmed the presence of viral antigen in epithelium of the stomach, colon, cecum, and ileum.^53^^,^^95^^,^^96^ Notably, intestinal lesions were frequently localized in areas rich in gut-associated lymphoid tissue (GALT), with viral antigen and cytopathic changes appearing in GALT prior to the surrounding mucosa, indicating a role of immune cells in MPXV infection of the digestive tract.^53^

When comparing MPXV clades, GI manifestations appear to be least common during infections with clade IIb, the lineage responsible for the 2022 outbreak, compared to clades IIa and I.^165^ This clinical observation is supported by findings in NHPs, where severe digestive tract lesions were observed following clade I infection, but not with clade II.^14^ Clade-specific differences in GI tropism have also been demonstrated *in vitro*. Infections of iPSC-derived colon organoids resulted in significantly lower levels of MPXV DNA and mRNA upon infection with clade IIb compared to clades I and IIa. Notably, clade I infection triggered a transcriptional downregulation of genes involved in zinc homeostasis, along with upregulation of markers linked to intestinal inflammation and apoptosis, such as cleaved caspase-3, indicating apoptosis and intestinal dysfunction.^78^ These findings were reinforced by a recent preprint study using organoids derived from human small intestine and rectal tissue. The authors reported increasing levels of viral DNA and infectious titers over time, confirmed intracellular virions via TEM, and observed widespread apoptotic cell death indicated by caspase-3 activation, organoid shrinkage, and epithelial disruption. Productive viral replication occurred mainly in enterocytes and goblet cells, whereas enteroendocrine cells appeared non-permissive.^173^

Closely associated with the GI tract, the liver has also emerged as a potential target organ during MPXV infection. Although direct evidence in humans remains limited, clinical observations include hepatomegaly and elevated liver enzymes, such as alanine aminotransferase (ALT) and aspartate aminotransferase (AST), with higher transaminase levels correlating with increased mortality.^68^^,^^147^^,^^174^ MPXV infection of the liver has been confirmed by findings in severe mpox cases of immunocompromised patients demonstrating hepatocellular necrosis with detection of viral antigen and DNA in hepatic tissue. Additionally, MPXV virions could be visualized by TEM in hepatocytes of these patients.^73^ These findings are further supported by animal studies. In several mouse models, MPXV infection involved the liver, as demonstrated by the detection of viral antigen, DNA, and infectious virus.^80^^,^^81^^,^^90^^,^^91^^,^^94^^,^^157^ Consistently, MPXV-infected NHPs developed hepatic lesions with viral antigen predominantly localized in Kupffer cells and, to a lesser extent, in hepatocytes, and infectious virus could be isolated from liver tissue.^14^^,^^53^^,^^95^ It is important to note that liver injury during MPXV infection may also result from antiviral therapy, as illustrated in a small study where three patients treated with brincidofovir, an orthopoxvirus DNA polymerase inhibitor, developed elevated liver enzymes, ultimately leading to discontinuation of treatment.^153^ Despite these observations in clinical and animal studies, *in vitro* investigations specifically addressing MPXV infection of hepatocytes or liver organoids remain scarce, leaving aspects of liver involvement poorly understood.

Collectively, the evidence highlights the GI tract as a common and clinically relevant target of MPXV infection. GI involvement appears to occur across multiple transmission routes and viral clades, although its frequency and severity may vary depending on viral lineage, host immune status, and site-specific factors, such as mucosal immune tissue.

### Urogenital system

Recent mpox outbreaks have highlighted the urogenital tract as a key site of clinical involvement, largely due to the shift in transmission dynamics. Unlike earlier outbreaks, the 2022 outbreak was primarily driven by close physical and sexual contact, leading to anogenital lesions in up to 73% of cases.^57^^,^^59^^,^^60^^,^^65^^,^^66^^,^^70^^,^^87^^,^^175^^,^^176^^,^^177^^,^^178^^,^^179^^,^^180^ Similarly, clade Ib infections in adults were often transmitted through sexual contact, resulting in urogenital symptoms similar to those of clade IIb infections.^71^ In addition to anogenital lesions, urogenital manifestations such as urethritis and penile edema have been observed.^65^^,^^71^^,^^178^^,^^181^^,^^182^^,^^183^ Although rare, cases of acute urinary retention and acute kidney injury requiring hospitalization and medical intervention have been reported.^57^^,^^184^^,^^185^^,^^186^ Moreover, MPXV DNA has been detected in urine samples, raising the possibility of direct renal involvement.^82^^,^^98^^,^^99^^,^^169^^,^^187^^,^^188^ However, due to the lack of human kidney biopsies, direct infection of renal tissue remains unconfirmed.

While mouse models provide evidence for direct kidney infection by detection of viral antigen, DNA, and replicating virus,^80^^,^^81^^,^^90^ findings from NHP studies remain inconclusive. Cynomolgus monkeys infected with aerosolized MPXV showed no histopathological kidney lesions or antigen staining, although replication-competent virus was sporadically isolated from kidney tissue.^53^ Similarly, earlier studies reported isolation of MPXV from kidneys of multiple monkey species, both with and without clinical signs of disease.^2^^,^^189^ It is worth noting that nephrotoxicity may also result from antiviral treatment. Cidofovir, the active compound of brincidofovir used in severe mpox cases, is known to cause dose-limiting kidney failure, complicating the distinction between mpox-associated kidney injury and drug-induced damage.^190^^,^^191^ Recent *in vitro* studies have aimed to clarify the potential for direct kidney infection. Multiple kidney-derived cell lines, including from African green monkeys, rhesus monkeys, vervet monkeys, pig embryos, baby hamsters, rabbits, and also human kidney cells, are susceptible to MPXV infection.^192^ This was further supported by infection of iPSC-derived human kidney organoids. MPXV replication was confirmed by increased viral DNA, rising viral loads, immunohistochemical staining, and visualization of virions, demonstrating that human kidney organoids support the full life cycle of MPXV. Glomerular and proximal tubular structures were widely infected, accompanied by a loss of epithelial integrity. In contrast, the distal tubular structures showed only limited infection.^193^ Notably, infected organoids secreted virus, consistent with the detection of viral DNA in patient urine samples.^99^^,^^169^^,^^193^

Collectively, these findings suggest potential direct infection of renal tissue by MPXV, which may contribute to urogenital symptoms and kidney complications observed in patients.

## Host immune response and immune evasion

The host immune response is a major determinant of MPXV disease outcome. Like other orthopoxviruses, MPXV expresses a variety of immune evasion factors that interfere with critical aspects of the host response, such as cytokine signaling, apoptosis regulation, major histocompatibility complex (MHC) expression, and T cell activation,^194^ extensively reviewed in Shchelkunov et al.^26^; Young^195^; Yi^196^; Shchelkunov^197^; and Weaver and Isaacs.^198^

The innate recognition of MPXV is mediated by pattern recognition receptors (PRRs), which sense viral components and trigger interferon (IFN) responses. IFNs constitute the first line of defense, inducing interferon-stimulated genes (ISGs) that restrict viral replication and spread.^199^ But, MPXV has evolved multiple mechanisms to block PRR sensing and IFN induction at various stages. For example, several B-cell lymphoma 2 (BCL-2)-like proteins encoded by *A47R*, *B13R*, *P1L*, *C6R*, and *D11L* inhibit the detection of viral double-stranded RNA (dsRNA) by intracellular PRRs.^26^^,^^197^ Downstream IFN signaling is also targeted, including the key transcription factors interferon regulatory factor 3 (IRF3) and nuclear factor kappa-light-chain-enhancer of activated B cells (NF-κB). IRF3 is blocked by the MPXV protein B16, while NF-κB activation is antagonized by a large set of ankyrin-like genes (e.g., *J3L*, *D1L*, *D7L*, *D9L*, *O1L*, *C1L*, *B5R*, *B17R*, *N4R*, and *J1R*).^197^ Beyond intracellular signaling, MPXV encodes several soluble proteins that act as decoys for immune mediators. For instance, *B16R* and *B9R* encode soluble IFN-binding proteins that capture type I and II IFNs before they reach their cellular receptors.^197^ Other proteins encoded by *A41L*, *B14R*, *D6L*, *J2R*, and *J3R* act as cytokine or chemokine antagonists, thereby impairing immune cell recruitment and signaling.^1^ The MPXV gene *D14L* also encodes a secreted protein known as the mpox inhibitor of complement enzyme (MOPICE) or complement control protein (CCP). This protein inhibits complement activation, thereby limiting a pathway that connects innate and adaptive immunity.^200^

Another key mechanism by which orthopoxviruses evade the immune system is the regulation of apoptosis, a caspase-driven process that eliminates infected cells. MPXV genes such as *P1L* and *C7L* encode proteins that bind to host pro-apoptotic proteins, inhibiting apoptosis.^1^^,^^201^

MPXV also targets the cellular arm of the innate immune response. NK cells normally exert cytolytic activity against infected cells and produce cytokines that influence inflammation.^202^ In experimental models, NK cells have been shown to protect CAST/EiJ mice from lethal MPXV infection, highlighting their role in controlling MPXV infection.^203^ Studies in MPXV-infected NHPs demonstrated a strong accumulation of NK cells, yet their function was suppressed.^204^ One mechanism involves the viral MHC class I-like protein (OMCP), which binds to the NK cell receptor NKG2D, preventing killing of infected cells.^205^ Monocytes also play a pivotal role in MPXV infection. They expand rapidly and are recruited to sites of infection to eliminate infected cells. However, they are themselves susceptible to MPXV and serve as vehicles for viral dissemination.^107^

Beyond innate immunity, adaptive immune responses strongly shape disease outcome. An early humoral immune response can support viral clearance, limit dissemination, and reduce disease severity.^206^ However, MPXV also interferes with these mechanisms. For example, T cell responses are dampened by the inhibition of Tcell receptor-mediated activation via an MHC-independent mechanism.^207^ Additionally, the viral M2 protein binds B7 ligands, thereby subverting the costimulatory signals required for full T cell activation.^208^ Furthermore, the *B10R* gene encodes a protein that blocks intracellular trafficking of MHC class I molecules, which limits antigen presentation to cytotoxic T cells.^197^

In summary, MPXV employs a multi-layered immune evasion strategy, affecting nearly all arms of host defense, to support viral replication. While many of these mechanisms have been identified, further research is needed to clarify their respective contributions to viral fitness and disease outcome.

## Conclusion and outlook

While MPXV infection is typically characterized by cutaneous lesions, it is increasingly recognized as a multi-organ disease, particularly in immunocompromised individuals and severe cases. Clinical observations, supported by viral detection (Table 1), animal studies, and *in vitro* analysis, indicate that MPXV can infect a wide range of tissues, including the respiratory, GI, neurological, urogenital, lymphatic, and ocular systems, with varying degrees of severity (Figure 1). Despite this growing recognition, the underlying pathophysiology remains poorly understood.

**Table 1:** Overview of MPXV detection across various human tissues and clinical samples

Organ/Samples	Infectious virus	Virion visualization	Viral antigen	Viral DNA	Comorbidities^a^
Skin	✓	✓	✓	✓	HIV (treated)^82^^,^^83^^,^^155^^,^^209^^,^^210^ HIV (untreated)^83^ HIV (no data)^73^^,^^74^^,^^98^^,^^99^ Transplant recipient^73^^,^^74^ HIV negative^77^^,^^82^^,^^83^^,^^98^^,^^99^^,^^155^^,^^209^ No data^75^^,^^76^^,^^154^^,^^188^
Eyes	No data	No data	✓	✓	HIV (no data)^73^ Transplant recipient^73^
Brain	No data	No data	Negative	Negative	HIV (no data)^73^ Transplant recipient^73^
CSF	No data	No data	No data	✓	Healthy^112^
Heart	No data	No data	Negative	Negative	HIV (no data)^73^ Transplant recipient^73^
Lymph nodes	No data	No data	✓	✓	HIV (no data)^73^ Transplant recipient^73^
Spleen	No data	No data	✓	✓	HIV (no data)^73^ Transplant recipient^73^
Bone marrow	No data	No data	✓	✓	HIV (no data)^73^ Transplant recipient^73^
Lung	No data	✓	✓	✓	HIV (no data)^73^ Transplant recipient^73^
GI tract	No data	No data	✓	✓	HIV (no data)^73^ Transplant recipient^73^
Liver	No data	✓	✓	✓	HIV (no data)^73^ Transplant recipient^73^
Saliva/oropharyngeal swabs	✓	No data	No data	✓	HIV (treated)^82^^,^^83^^,^^152^^,^^155^^,^^209^ HIV (untreated)^83^ HIV (no data)^70^^,^^98^^,^^99^^,^^169^^,^^172^ Diabetes^70^ Crohn’s disease^70^ HIV negative^70^^,^^82^^,^^83^^,^^98^^,^^99^^,^^152^^,^^153^^,^^155^^,^^169^^,^^172^^,^^209^ No data^153^^,^^154^^,^^188^
Rectal swabs	✓	No data	No data	✓	HIV (treated)^82^^,^^83^^,^^155^^,^^168^^,^^170^ HIV (untreated)^83^^,^^168^ HIV (no data)^70^^,^^98^^,^^166^^,^^169^^,^^171^^,^^172^ Diabetes^70^ Crohn’s disease^70^ HIV negative^70^^,^^82^^,^^83^^,^^98^^,^^155^^,^^166^^,^^168^^,^^169^^,^^170^^,^^171^^,^^172^ No data^154^
Semen	✓	No data	No data	✓	HIV (treated)^82^^,^^83^^,^^155^^,^^210^ HIV (untreated)^83^ HIV (no data)^70^^,^^99^^,^^169^ Diabetes^70^ Crohn’s disease^70^ HIV negative^70^^,^^82^^,^^83^^,^^99^^,^^155^^,^^169^
Urine	No data	No data	No data	✓	HIV (treated)^82^^,^^209^ HIV (no data)^70^^,^^98^^,^^99^^,^^169^^,^^172^ Diabetes^70^ Crohn’s disease^70^ HIV negative^70^^,^^82^^,^^98^^,^^99^^,^^153^^,^^169^^,^^172^^,^^209^ No data^188^
Stool	✓	No data	No data	✓	HIV (treated)^155^ HIV (no data)^99^^,^^169^ HIV negative^99^^,^^155^^,^^169^
Blood (whole blood, serum or plasma)	Negative	No data	No data	✓	HIV (treated)^82^^,^^83^^,^^155^^,^^209^ HIV (untreated)^83^ HIV (no data)^70^^,^^98^^,^^99^ Diabetes^70^ Crohn’s disease^70^ HIV negative^70^^,^^82^^,^^83^^,^^98^^,^^99^^,^^155^^,^^209^ No data^153^^,^^188^

The presence of infectious virus, virions (by electron microscopy), viral antigens, and viral DNA is indicated.

✓, detected; CSF, cerebrospinal fluid.

a As stated in the reviewed literature.

Much of our knowledge on MPXV dissemination in humans comes from immunocompromised patients, who are over-represented among severe and fatal cases (Table 1). Within this group, people living with HIV are particularly relevant, given that HIV prevalence is high in MPXV-endemic regions, reaching up to ∼10%.^211^ Moreover, during the 2022 outbreak, the majority of mpox cases occurred among men who have sex with men, of whom 36%–67% were HIV positive.^19^ Uncontrolled HIV infection with low CD4^+^ T cell counts has been associated with more severe disease manifestations, including extensive and necrotizing skin lesions, pulmonary involvement, secondary infections, prolonged illness, sepsis, and higher mortality.^56^^,^^85^^,^^167^ In contrast, when HIV infection was well controlled, as in most patients during the 2022 outbreak, the clinical presentation and disease severity were largely comparable to HIV-negative individuals.^57^^,^^59^^,^^60^ These observations point to a critical role of CD4^+^ T cells in controlling MPXV infection and underscore the need for further research to determine how HIV status and other forms of immunosuppression influence MPXV pathogenesis.

Evidence from severe and fatal mpox cases in immunocompromised patients suggests that tissue damage stems from uncontrolled MPXV replication resulting in tissue necrosis and infarction.^73^^,^^212^ *In vitro* studies further support this, demonstrating MPXV-induced cell death in the absence of strong inflammatory responses.^122^^,^^163^ These findings point to direct cytopathic effects as a key driver of organ damage in severe disease.

Further research is needed to elucidate the mechanisms that mediate the broad host and tissue tropism of MPXV. To date, no specific cellular receptor for MPXV has been identified. Instead, the virus appears to utilize conserved, ubiquitously expressed surface components for attachment and entry.^29^^,^^30^^,^^31^^,^^32^ Consequently, productive viral replication likely depends on intracellular processes following viral entry.^44^^,^^45^^,^^46^

The ability of viruses to establish productive replication is further influenced by the host immune response, which, in turn, is counteracted by viral immunomodulatory proteins.^213^ Interestingly, comparative genomic analyses have revealed genetic differences between MPXV clades, which likely contribute to the observed variation in virulence, immune evasion, and tissue tropism. These differences are especially pronounced in genes encoding immune-modulatory proteins. For example, the *D14L* gene encoding for MOPICE is present in clade I but absent in clade II, potentially leading to a higher susceptibility of clade II to inactivation by the complement system.^214^^,^^215^ Notably, recent findings suggest that the newly emerged clade Ib may also lack the gene encoding for CCP.^216^^,^^217^ In addition, clade I encodes a full-length version of the BR-203 virulence factor, implicated in regulation of apoptosis, whereas clade II harbors a truncated form that may diminish its regulatory capacity.^198^

Moreover, recent findings indicate that different MPXV clades elicit varying degrees of immunogenicity, leading to clade-specific differences in host immune responses and disease outcomes. This is supported by studies in CAST/EiJ mice, which showed that infection with clade IIb triggered a faster and more robust immune response than clade IIa, resulting in less severe disease.^94^

Overall, these insights highlight that MPXV is not merely a dermatotropic virus but a complex pathogen capable of systemic dissemination and multi-organ involvement, particularly in vulnerable populations. The extent and pattern of organ involvement in mpox appear to be influenced not only by host immune status but also by clade-specific viral factors, which may account for differences in disease severity, tissue tropism, and clinical presentation observed across MPXV lineages. However, the mechanisms driving MPXV pathology across different organ systems remain poorly understood. Future research should focus on elucidating the determinants of viral tropism, especially in less commonly affected but clinically significant organs, such as the heart and liver, to better understand the drivers of severe disease. Moreover, further efforts are required to dissect the mechanisms underlying clade-specific differences in clinical presentation and disease severity, particularly in the context of the ongoing resurgence of MPXV clade Ib. A deeper understanding of MPXV biology and host interactions is essential not only for guiding the development of targeted therapeutics and vaccines, but also for improving outbreak preparedness and global health responses to future outbreaks.

## Acknowledgments

We thank all members of the Institute for Infection Research and Vaccine Development for helpful suggestions and discussions. Figures were created using BioRender. T.L.M. was funded by the German Center for Infection Research (10.13039/100009139DZIF, TTU 01.719). T.L.M. and J.S.z.W. are associated with or funded by the 10.13039/501100001659DFG collaborative research center 1648 (SFB 1648/1 2024–512741711). J.S.z.W. is also funded by 10.13039/100009139DZIF.

## Author contributions

A.L.-R., investigation and writing – original draft preparation; J.P.M, visualization and writing – review & editing; J.S.z.W., writing – review & editing; T.L.M., conceptualization, supervision and writing – review & editing

## Declaration of interests

The authors declare no conflict of interest.

## Declaration of generative AI and AI-assisted technologies in the writing process

During the preparation of this work the authors used ChatGPT in order to improve grammar and perform minor language editing. After using this tool/service, the authors reviewed and edited the content as needed and take full responsibility for the content of the publication.
